# Racemic mimics. Part 1. Nickel coordination com­pounds

**DOI:** 10.1107/S2053229625001147

**Published:** 2025-03-06

**Authors:** Ivan Bernal, Roger A. Lalancette

**Affiliations:** a Carl A. Olson Memorial Laboratories, Department of Chemistry, Rutgers University, 73 Warren St, Newark, NJ, USA; b Molecular Sciences Institute, School of Chemistry, University of the Witwatersrand, Private Bag 3, 2050 Johannesburg, South Africa; Rigaku Americas Corporation, USA

**Keywords:** racemic mimics, coordination com­pounds, nickel amines, L-leucine ligands, Sohncke space groups, Z′ = 2.0, crystal structure

## Abstract

To date there are very few examples of crystallographically well-documented racemic mimics. The original discovery of this class of crystals occurred at a time when crystallography was in its infancy, data collection and processing were tedious and limited by X-ray equipment, and com­puting power was indeed limited. Therefore, this inter­esting class of crystalline mol­ecules, potentially having useful biological uses, is today one of those scientific orphans largely ignored in the crystallographic realm. As proof of this, to date, you cannot systematically search for this class in databases. Thus, for the time being, there are few satisfactory examples of high-quality crystal structures of both members of such pairs which have been highlighted in the literature. Finally, being largely undocumented, there are no useful clues to guide you as to how to guess the classes of com­pounds likely to produce such pairs. The question then is, how do we go about searching for potential cases of such crystallization modes using information already in print? Herein, we provide some suggestions we believe are useful, and to the extent possible with such data, to illustrate the possibilities offered by such an approach.

## Abstract 2

There are very few examples of crystallographically well-documented racemic mimics. Therefore, this inter­esting class of crystalline mol­ecules, potentially having useful biological uses, is today one of those scientific orphans largely ignored in the crystallographic realm. Some suggestions are provided for searching for potential cases of such a crystallization mode using information already in print.

## Introduction

Previously (Wood *et al.*, 2021 ▸), we briefly dealt with examples of crystals belonging to the category of *racemic mimics*, which thus far is not a widely-known phenomenon, despite the fact that the published literature appears to contain large numbers of examples of species belonging to that class, as will be demonstrated. Thus, we present a brief introduction to the concept, taken from a published article (Wood *et al.*, 2021 ▸), that we paraphrase below (edited), because it was succinct and is appropriate for this discussion.

‘For a clear and concise description of an early awareness of the existence of this type of crystalline material, we refer the reader to four seminal papers constituting some of the earliest modern descriptions of what a *racemic mimic* substance is.’ These papers are, in historical order, by Furberg & Hassel (1950 ▸), who studied the crystal structure of phenyl glyceric acid grown slowly from water; Schouwstra, who studied crystals of dl-methyl­succinic acid grown by sublimation (Schouwstra, 1973*a* ▸) and from water solution (Schouwstra, 1973*b* ▸); and Mostad *et al.* (1975 ▸), who examined *o*-tyrosine crystals grown from methanol containing small amounts of ammonia to increase its solubility. In all cases, the crystals of both the *racemate* and the *optically-pure material* crystallized with nearly identical unit-cell constants; consequently, while the value of *Z*′ was 1.0 for the racemic samples, the value for the pure enanti­omers was 2.0, and, while the lattice of the former contains racemic pairs, the latter contained *pairs of pure enanti­omers*. Thus, they asked: ‘why, and how?’ In a remarkably clear and simple answer, Furberg & Hassel (1950 ▸) indicated that the pure chiral material seemed to crystallize ‘*as if a twin resembling in its packing that of the true racemate*’: in other words, as a ‘racemic mimic’; thus the name. They also proposed that substances containing flexible (dissymmetric) fragments whose torsional barriers were low would make ideal candidates for the existence of such a phenomenon, and they documented additional cases. That was a remarkably ad­vanced concept for its day – and happens to conform to what we describe in this article, since we already had an example of a *racemic mimic* in the case of the cocaine derivative of Erdmann’s salt (Wood *et al.*, 2021 ▸).

At this point it is worth remarking that two of the most authoritative and highly informative treatises in crystal chemistry do not mention this topic in even their indices. They are: (*a*) ‘*Polymorphism in Mol­ecular Crystals*’ (Bernstein, 2002 ▸) and (*b*) ‘*Enanti­omers, Racemates and Resolutions*’ (Jacques *et al.*, 1981 ▸), this being a remarkable state of affairs since examples of material closely related to this topic are discussed on p. 19 of Jacques *et al.* (1981 ▸) (see Fig. 10 therein).

Unfortunately, we are unable to use the original examples (Furberg & Hassel, 1950 ▸; Schouwstra, 1973*a* ▸, 1973*b* ▸; Mostad *et al.*, 1975 ▸) to illustrate the packing similarities and differences in a racemic mimic pair of crystal structures because those ancient data sets are incom­plete, most importantly, due to missing H atoms in their atom lists, and those atoms are key to the packing of such substances. As for Wood *et al.* (2021 ▸), we inferred that the cocaine derivative in question was a racemic mimic because the packing diagram described displays the required characteristics from the Sohncke member of the *expected* racemic mimic pair we postulated in that article (Wood *et al.*, 2021 ▸).

## Searching the CSD

Given the appearance of a case of racemic mimic crystallization in our own work (Wood *et al.*, 2021 ▸), we decided to investigate the contents of the Cambridge Structural Database (CSD; Groom *et al.*, 2016 ▸) using the program *Mercury* (Macrae *et al.*, 2020 ▸) to determine the chirality appropriate to the constituents of such pairs present in each case and, finally, to generate appropriate figures using *DIAMOND* (Putz & Brandenburg, 2016 ▸).

The authors of the previously quoted material (Furberg & Hassel, 1950 ▸; Schouwstra, 1973*a* ▸, 1973*b* ▸; Mostad *et al.*, 1975 ▸) were focused on the novel observation that a pure chiral substance, such as an amino acid, can crystallize in a Sohncke space group with *Z*′ = 2 in a lattice that is, for all practical purposes, identical with that of its racemate with *Z*′ = 1; this observation can be reworded as follows: ‘while very closely retaining the values of the unit-cell constants of racemic crystals of com­pound *X* (*Z*′ = 1), its pure chiral analog crystallizes in a *Sohncke subgroup* of that with *Z*′ = 2.’ Questions: (*a*) what classes of com­pounds can do that?; (*b*) what are the space groups in which this happens?; (*c*) what chiral properties are shared or are different in the com­ponents of *Z*′ = 2?; (*d*) within classes of compounds (*e.g.* octahedral *versus* planar), are there portions of molecules which play a more important role in the probability of producing a racemic mimic?

The procedure used was: search the CSD for a specific metal (for example, any transition metal) and specify that the mol­ecule must crystallize in a Sohncke space group with *Z*′ = 2, only. The resulting list was then searched for CSD-provided comments that the racemate had also been studied for which, fortunately, the relevant refcode is provided. Those pairs were then included in our list if the criteria of reliability, for both, was deemed attractive: for example, demand that *R* ≤ 5.0%, no errors, *etc*. Whenever a structure was determined at more than one tem­per­a­ture, the one at lowest tem­per­a­ture was chosen if the other criteria were met equally, or approximately so. When such a pair (enanti­omer and racemate) was found, the two enanti­omeric species were matched in *Mercury* (Mol­ecule Overlay), at which point a selection was made of the output model to be used for display in the manuscript, usually ‘inversion’ or ‘flexibility’. Finally, the packing was ex­amined for evidence of *pseudo-inversion centers* using *DIAMOND*. Please see Appendix *A*[App appa] for a step-by-step description of the process we followed in generating the figures in *Mercury*.

We succeeded in the case of the pair **IHEKIP** (Calmuschi & Englert, 2002 ▸) and **BOHBIK** (Calmuschi-Cula *et al.*, 2009 ▸), as an example, albeit that the metal here is palladium, and not nickel. The characterizations are as follows:

**IHEKIP**: (*SP*-4-4)-(*R*)-[2-(1-amino­eth­yl)phen­yl]chloro­py­ri­dine­palladium(II), C_13_H_15_ClN_2_Pd, space group *I*4_1_, *Z* = 16.0, *Z*′ = 2.0, *R* = 4.60% and *T* = 293 K.

**Chirality data**: Pd1(*S*) and C7(*R*); Pd2(*R*) and C20(*R*).

**BOHBIK**: (*SP*-4-4)-[*rac*-2-(1-amino­eth­yl)phen­yl]chloro­py­ri­dine­palladium(II), C_13_H_15_ClN_2_Pd, space group *I*4_1_/*a*, *Z* = 16.0, *Z*′ = 1.0, *R* = 3.76% and *T* = 100 K.

**Chirality data**: a racemate.

A com­parison of the unit-cell constants can be found in Table 1 ▸.

**Figure 1:**
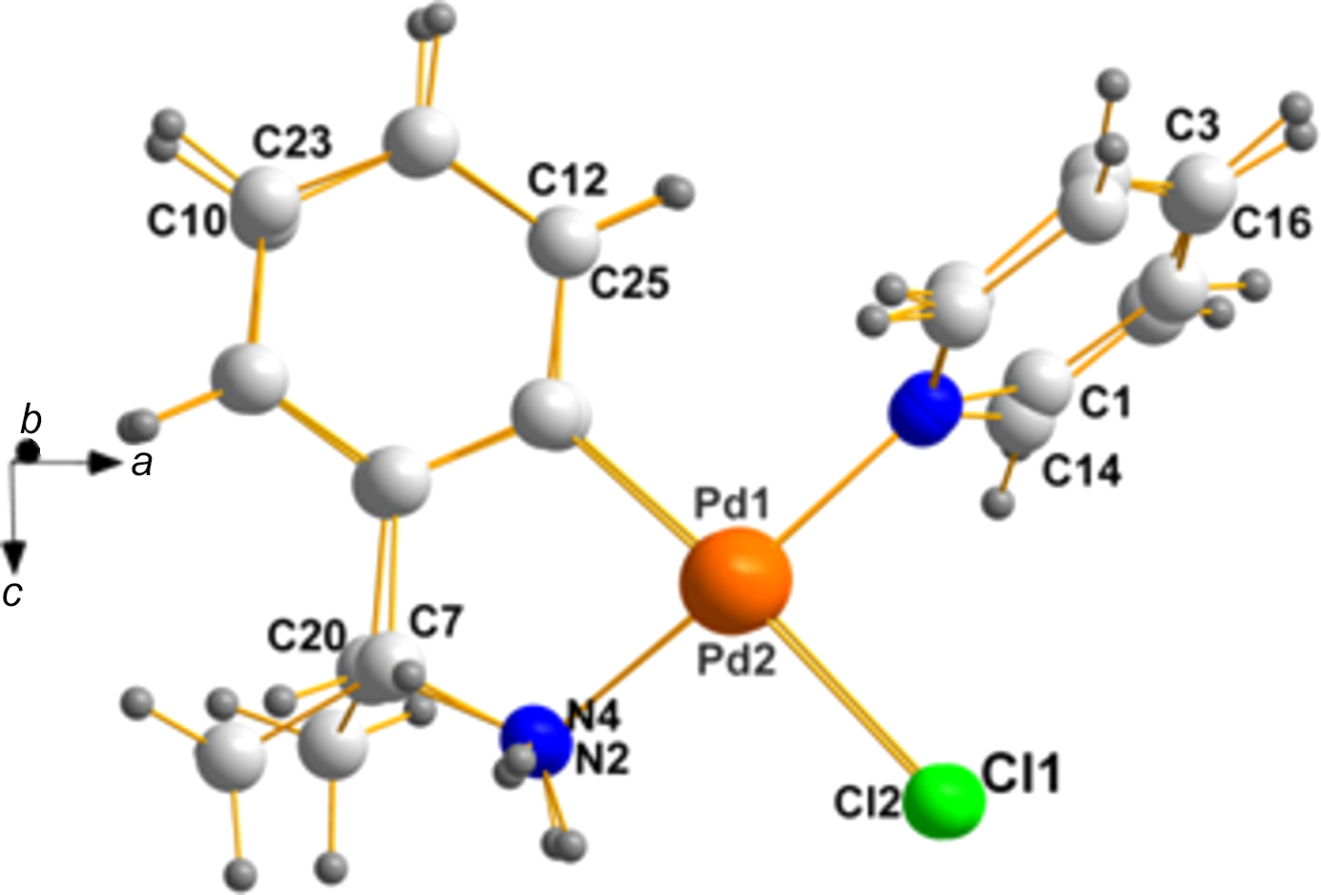
A superposition of the two mol­ecules of the same chirality in **IHEKIP**. The chiral C atoms in the two mol­ecules of the asymmetric unit in **IHEKIP** are C7 and C20, shown lower left.

**BOBHIK** contains identical pairs demanded by space-group symmetry. Therefore, it is of inter­est to com­pare the independent members of the enanti­omeric pair present in **IHEKIP** to determine to what extent their geometry agrees (disagrees). We accom­plished this by the use of *Mercury*, as shown in Fig. 1 ▸, which was generated with *DIAMOND*.

An important symmetry element distinguishing centrosymmetric (racemic) structures from those of their racemic mimic counterparts is that the center of mass of the former must be exactly at an inversion center such as (0.0000, 0.0000, 0.0000) or (0.2500, 0.2500, 0.2500), *etc.*, whereas those associated with the latter are somewhat similar, but slightly imperfect, as, for example, with (0.4972, −0.0023, 0.5033), *etc*. That is an excellent clue to look for when searching for potential cases of racemic mimics in the contents of the CSD.

**Note**: it is important to recall that in the monoclinic system, such as in the case of *P*2_1_, the origin in *y* is totally arbitrary and can be set, for all practical purposes, at 0.0000.

We now proceed to describe a group of four nickel coordination com­pounds whose structures are not available in both enantiopure and racemic form, but which are available in attractive precision and which, like **IHEKIP** (Schouwstra, 1973*a* ▸), belong in the former class with *Z*′ = 2 and are likely to have racemic counterparts. That is the aim of this article, *i.e.* to provide a clue as to which coordination com­pounds are likely to produce racemic counterparts.

## Results of the CSD search and discussion of the nickel com­pounds

Throughout, the identification of the chiral centers was carried out with *Mercury* and the Centers of Mass were com­puted with *DIAMOND*, using the routine ‘View’. Note that the same center of mass calculation can be carried out using *Mercury*.

### Example 1

**NOWPIA**, space group *P*2_1_; {(*S*)-[(2-{[(*S*)-1-benzyl­prol­yl]amino}­phen­yl)(phen­yl)methyl­ene]amino}[(*S*)-(3-oxo-2,3-di­hy­dro-1*H*-isoindol-1-yl)acetato]­nickel(II) methanol solvate (Li *et al.*, 2015 ▸).

**Chirality data**: Ni1(*R*), N3(*R*), C11(*S*), C2(*R*), and C28(*S*); Ni2(*R*), N7(*R*), C46(*S*), C37(*R*), and C63(*S*).

**Center of mass**: 0.0103, 0.0219, 0.009; note how close it is to 0.0000, 0.0000, 0.0000.

**Figure 2:**
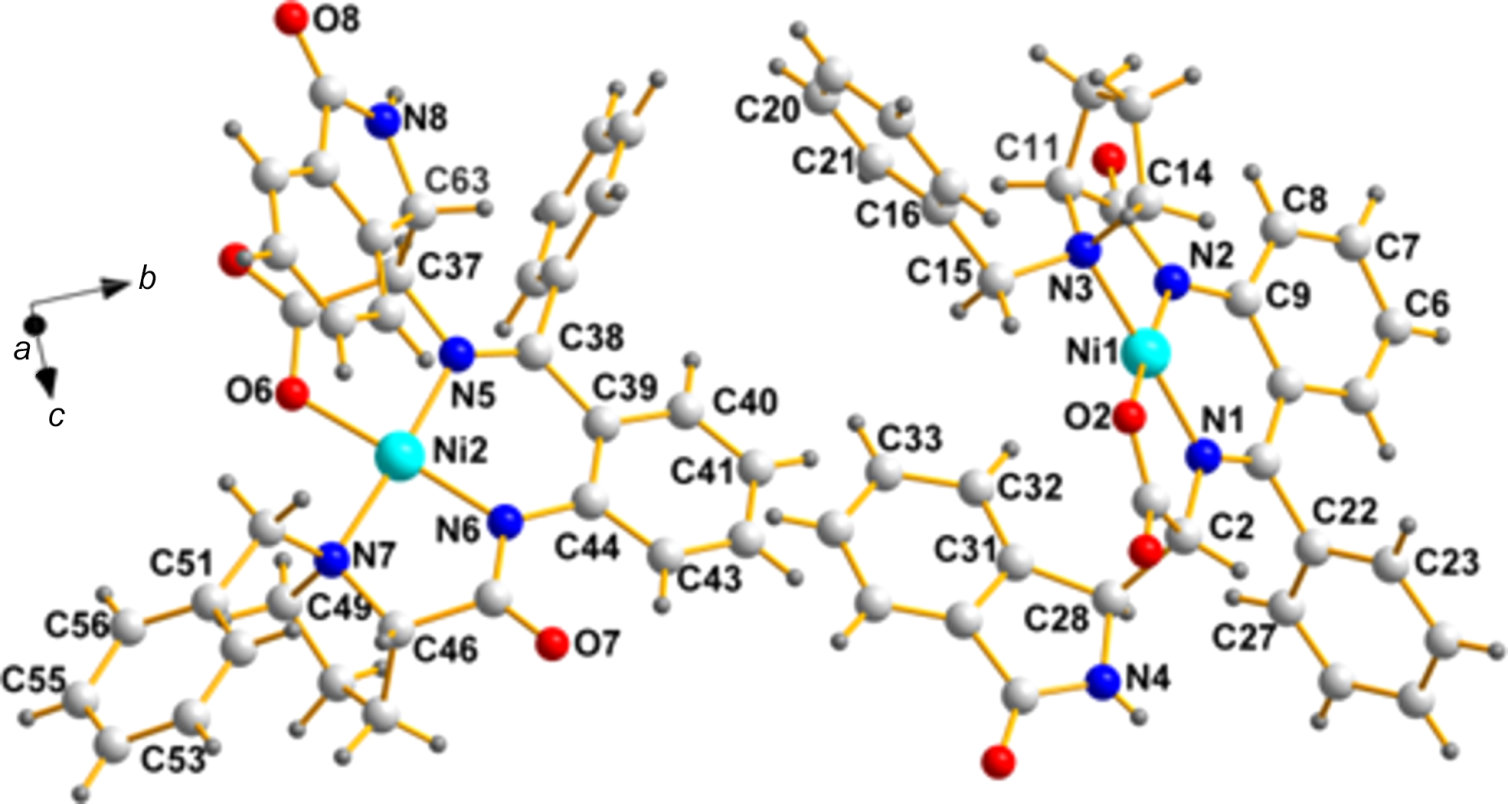
A view of the two mol­ecules in the asymmetric unit of **NOWPIA**, showing the labels of the atoms, including the chiral centers (N3, C11, C2, and C28; and N7, C46, C37, and C63).

Fig. 2 ▸ depicts the pair present in the asymmetric unit and most of the atom labels needed for com­parison of their stereochemistry, specifically, the chiral C atoms.

**Figure 3:**
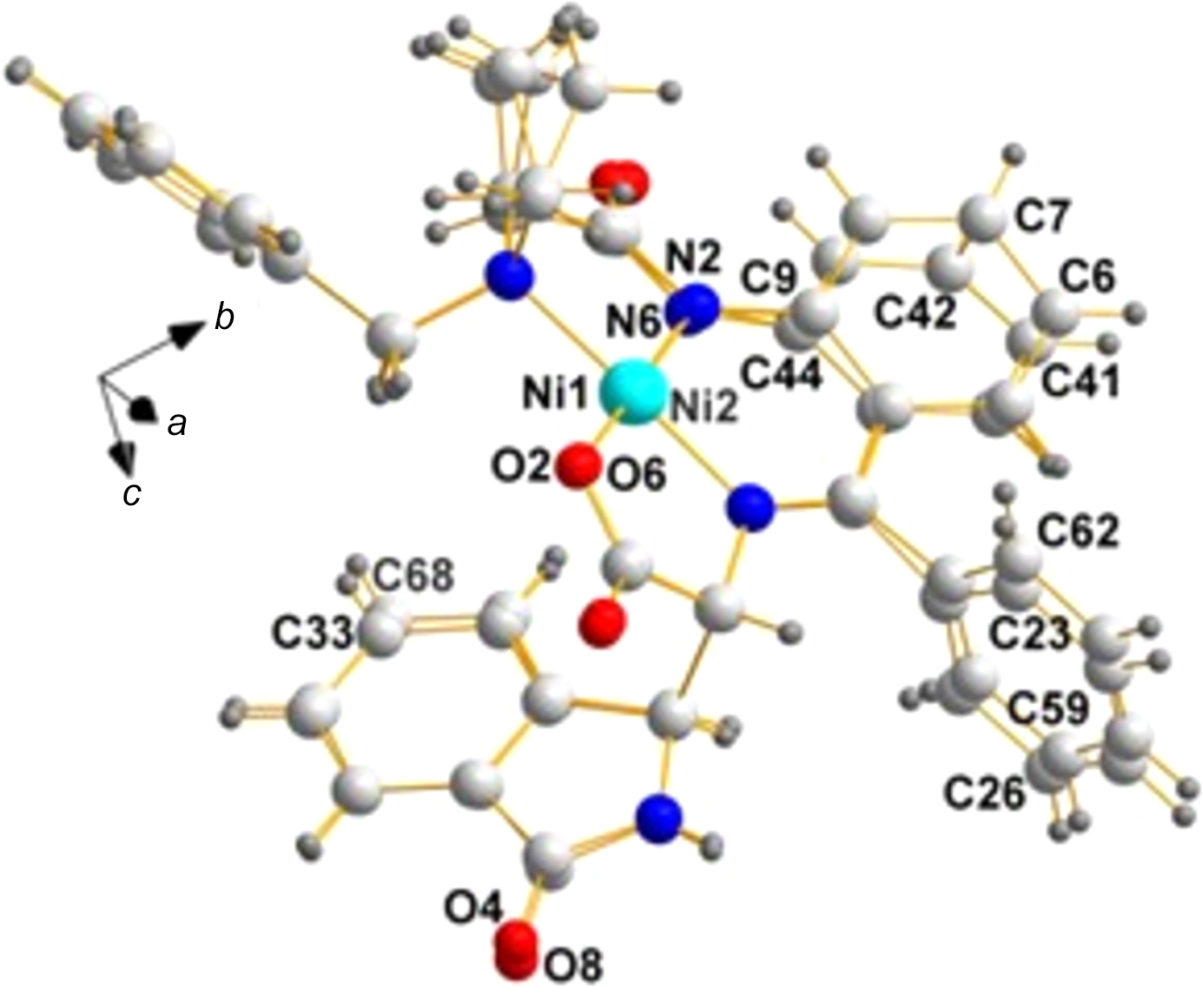
A superposition of the two mol­ecules of the same chirality in **NOWPIA**. The extent to which these two mol­ecules match is impressive, especially since considerable substituents are attached to the central Ni atom(s) by single bonds.

An inter­esting way to visualize the extent to which the two mol­ecules in the asymmetric unit agree or disagree is to examine them with the ‘overlay’ option in *Mercury* (see Fig. 3 ▸). In generating the overlapped results, the only command used was ‘flexibility’.

### Example 2

**WALJOK**, space group *P*2_1_; (*R*)-[({2-[1-(3-fluoro­benz­yl)pyrrolidine-2-carboxamide]­phen­yl}phenyl­methyl­ene)alaninato-κ^4^*N*,*N*′,*N*′′,*O*]nickel(II) chloro­form solvate hemihydrate, (*R*)-{*N*-[(2-{[1-(3-fluoro­benz­yl)prol­yl]amino}­phen­yl)(phen­yl)me­thyl­ene]alaninato-κ^4^*N*,*N*′,*N*′′,*O*}nickel(II) chloro­form solvate hemihydrate (Saghyan *et al.*, 2010 ▸).

**Chirality data**: Ni1(*R*), C2(*R*), N3(*S*), and C11(*R*); Ni2(*R*), C30(*R*), N6(*S*), and C39(*R*).

**Center of mass**: 0.0386, −0.0205, 0.4970; note how close it is to 0.0000, 0.0000, 0.5000.

**Figure 4:**
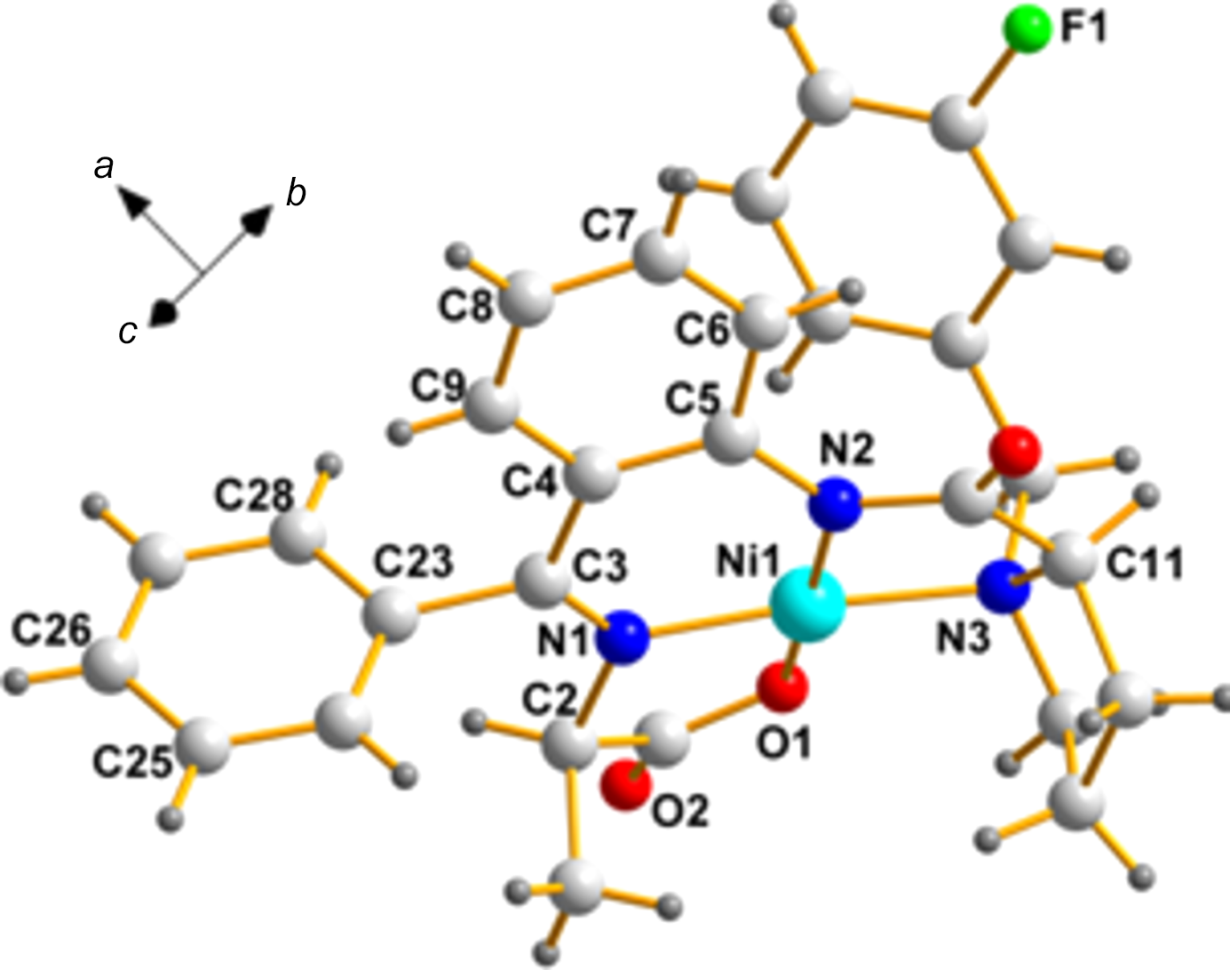
This view of **WALJOK** was chosen to display chiral atoms C2, N3, and C11 as clearly as possible. Unfortunately, both mol­ecules could not be shown equally clearly in a single image.

**Figure 5:**
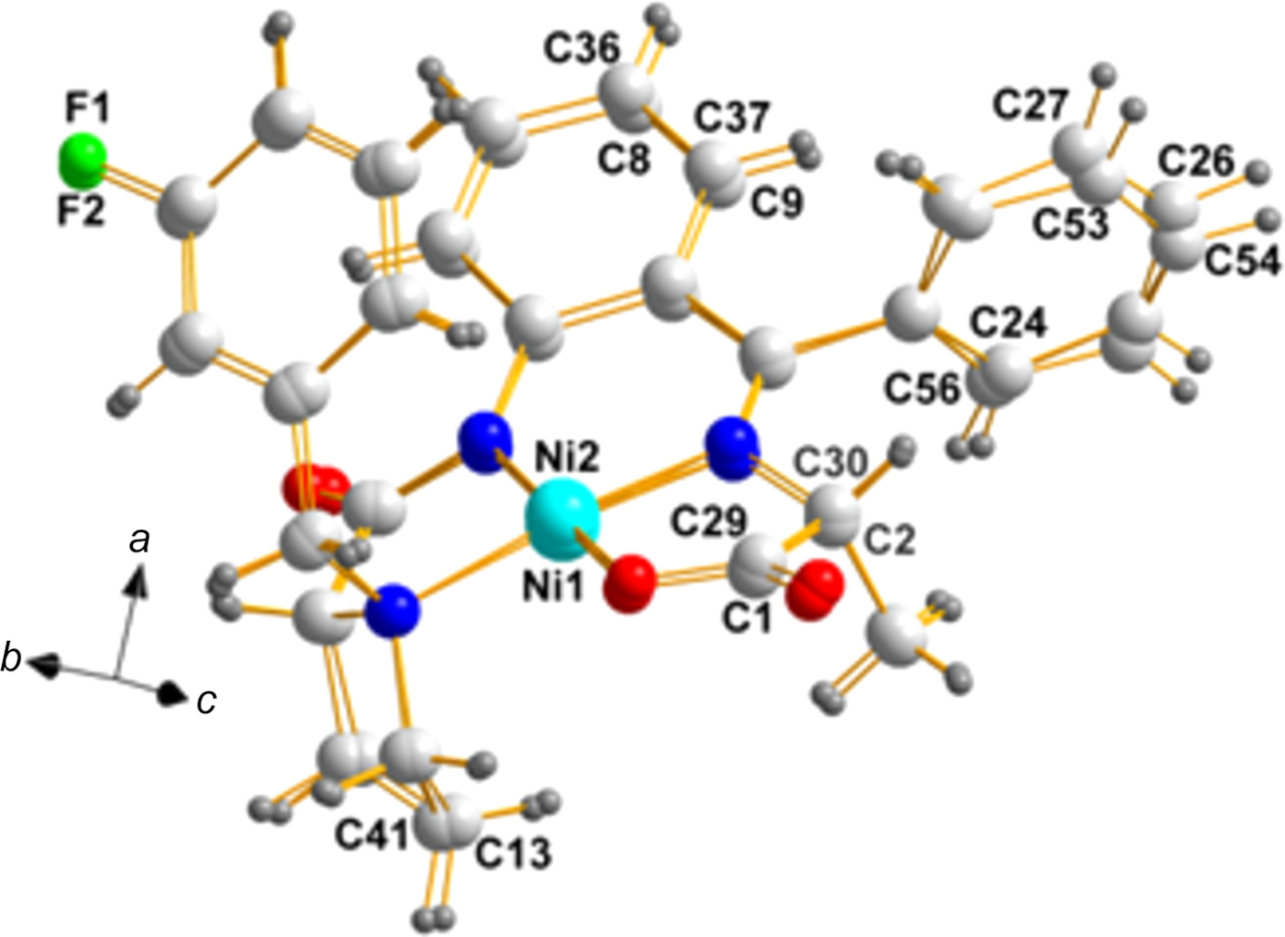
A superposition of the two mol­ecules of the same chirality in **WALJOK**. This pair of mol­ecules displays the labels of overlapping atomic pairs, such as the chiral C2 (mol1) and C30 (mol2) atoms. Given the necessary size of the labels, there was no room to conveniently display the labels of the other two pairs (N3/N6 and C11/C39).

Because it was very difficult to obtain a single view de­picting both diastereoisomers while avoiding excessive over­lap of important fragments, in Fig. 4 ▸ we show Ni1 only. The fact that the two mol­ecules are matched very closely in stereochemistry is made obvious in the overlay picture in Fig. 5 ▸.

**Figure 6:**
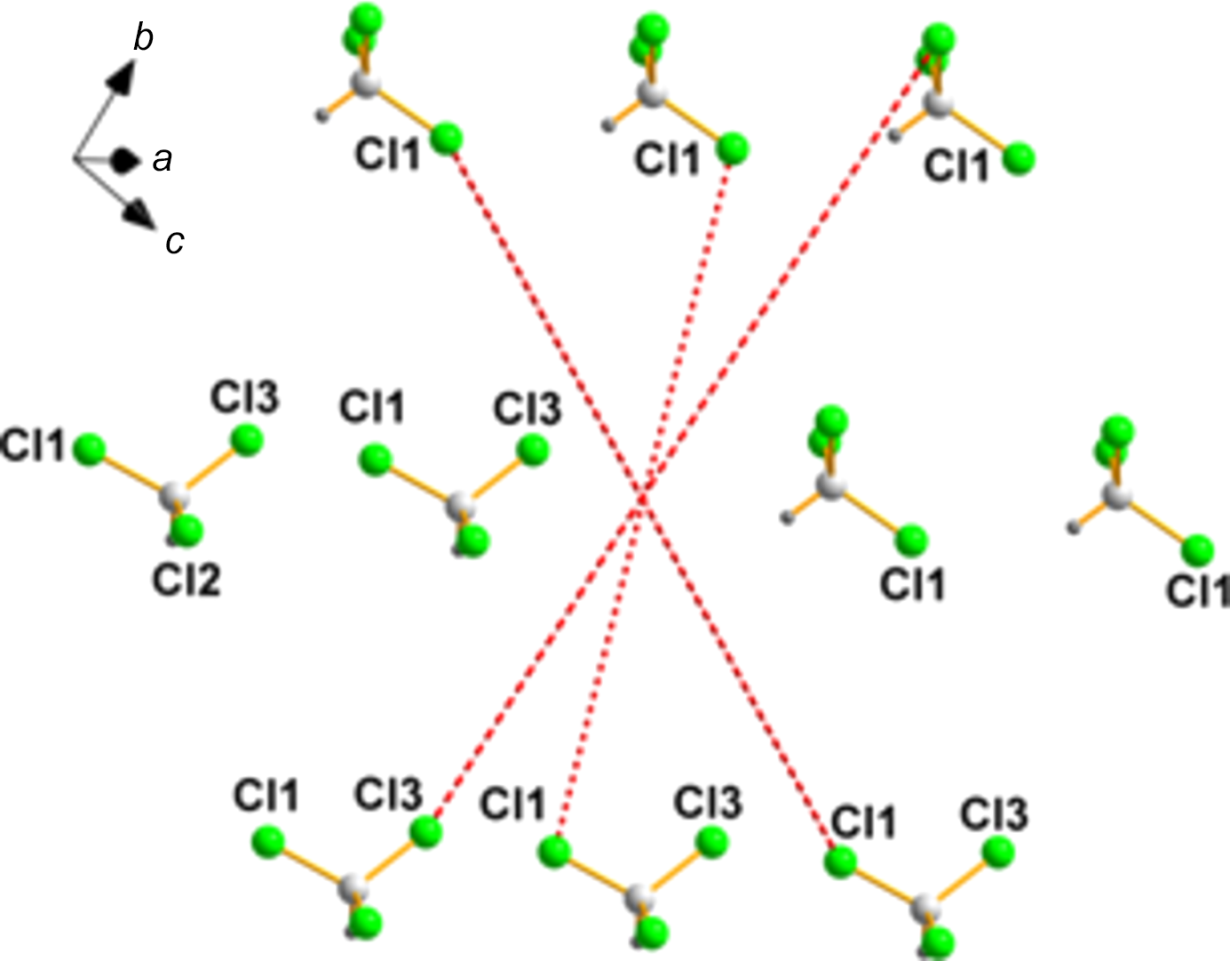
One pseudo-inversion center (located at 0.0000, 0.7209, 0.0000, which is very close to 0, 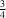, 0) is denoted by the inter­section of the dotted red lines that connect not only those pairs selected, but also additional ones.

The packing of the mol­ecules in the lattice of **WALJOK** is such that the overall center of mass is located near a pseudo-inversion center (located at 0.0000, 0.7209, 0.0000, which is very close to 0, 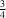, 0) shown in Fig. 6 ▸.

The reader can easily note that quartets of chloro­form mol­ecules, such as those upper left and right, as well as lower left and right, also share additional pseudo-inversion centers that are spread throughout the entire three-dimensional lattice. Additional comments on the effect of such features are given in the *Conclusions*.

### Example 3

**ZAGZUF**, space group *P*2_1_2_1_2_1_; [*N*-({2-[(1-benzyl­prol­yl)amino]­phen­yl}(phen­yl)methyl­ene)-4-fluoro-β-methyl­phenyl­alaninato]nickel(II) (de Meijere *et al.*, 2014 ▸).

**Chirality data**: Ni1(*S*), C3(*S*), N2(*R*), C27(*R*), and C31(*S*); Ni2(*S*), C40(*S*), N5(*R*), C38(*R*), and C44(*S*).

**Center of mass:**: 0.5055, 0.5011, 0.5014; note how close it is to 0.5000, 0.5000, 0.5000.

**Figure 7:**
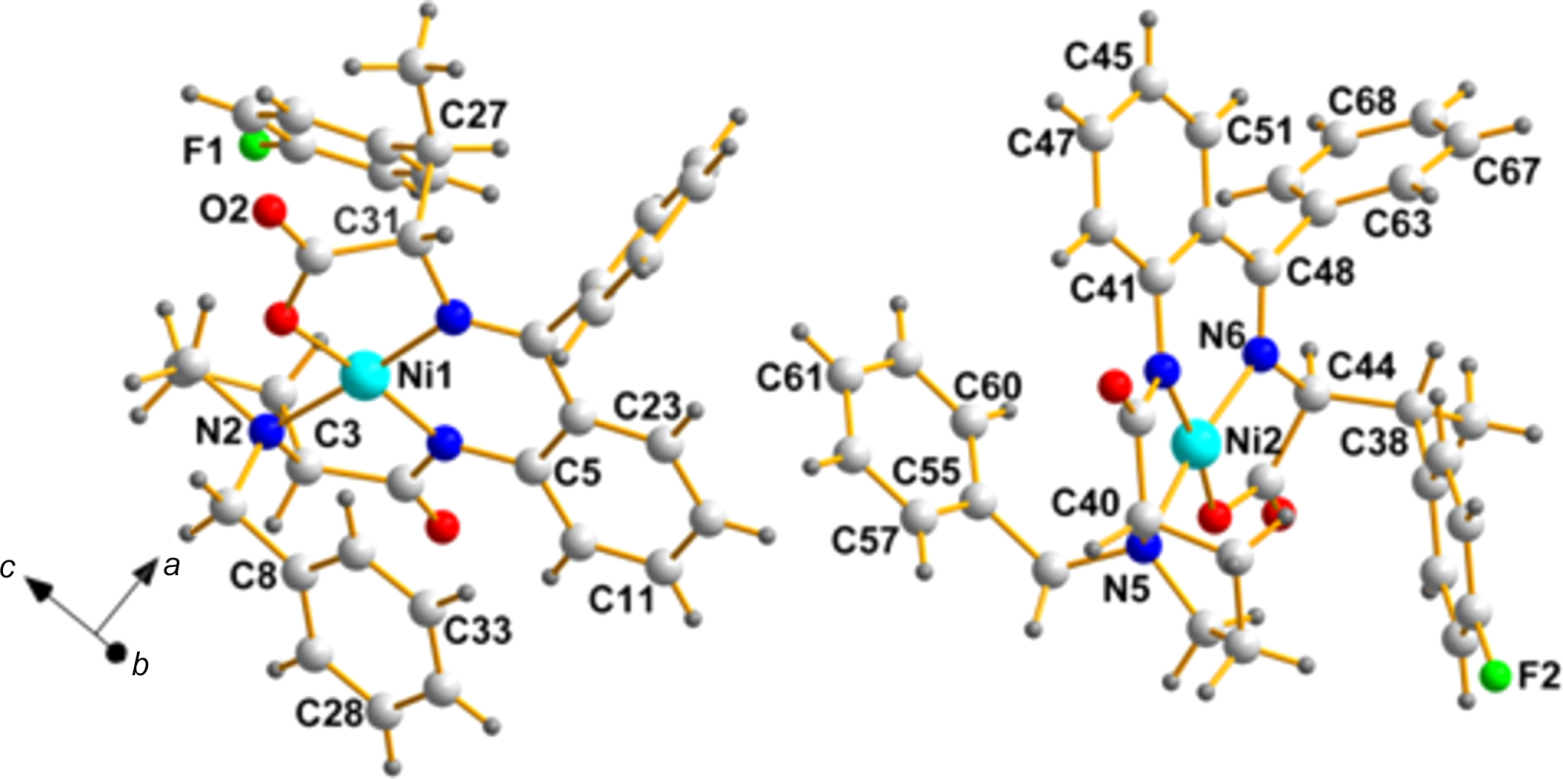
A view of the two mol­ecules in the asymmetric unit of **ZAGZUF** (de Meijere *et al.*, 2014 ▸), with labels identifying the chiral center pairs listed above (C3/C40, N2/N5, C27/C38, and C31/C44).

**Figure 8:**
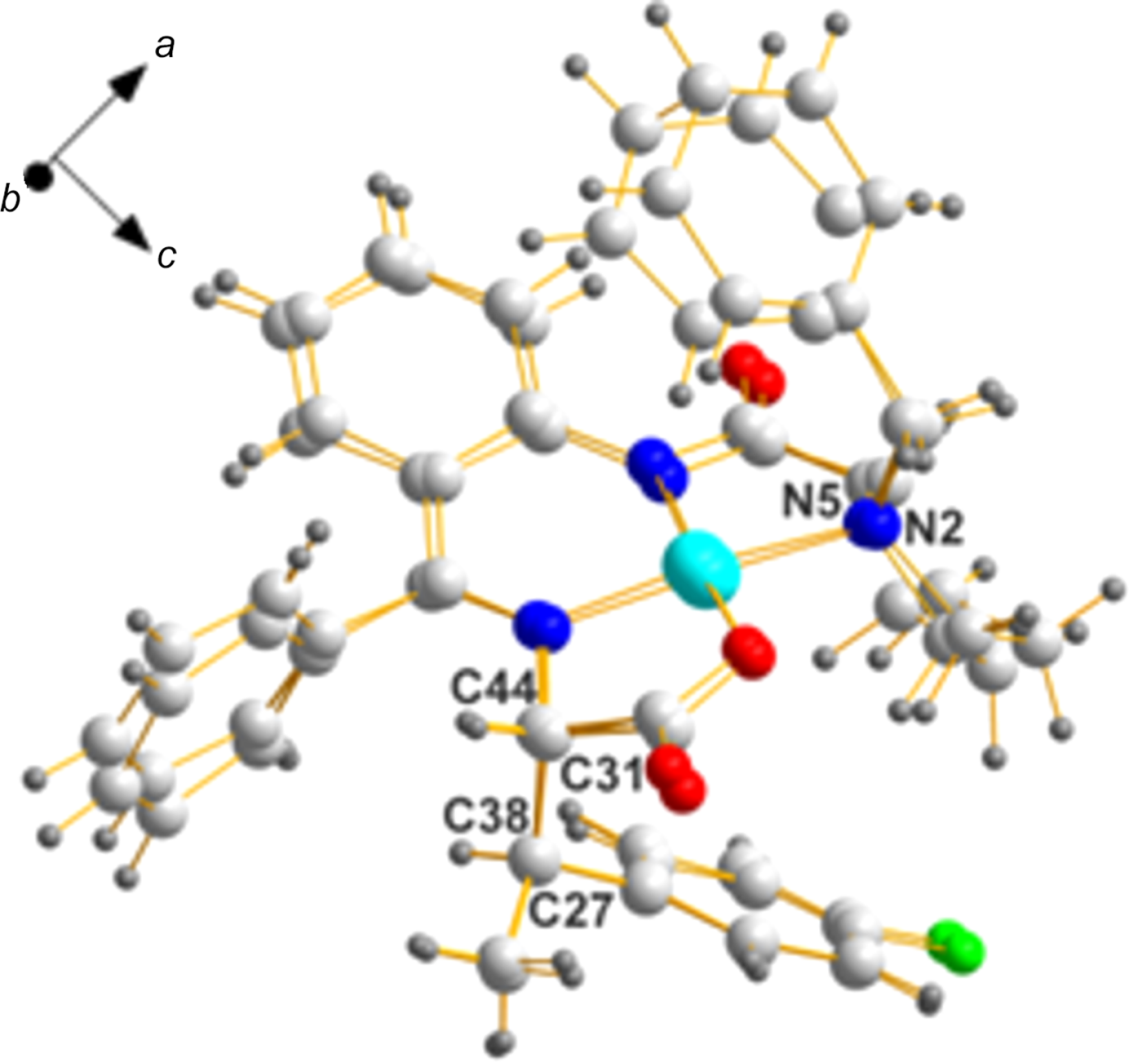
A superposition of the two mol­ecules of the same chirality in **ZAGZUF**. The degree of overlap is remarkable for such a flexible mol­ecule. Only three of the four (C3/C40, N2/N5, C27/C38, and C31/C44) chiral center pairs are shown.

In Fig. 7 ▸, we illustrate the mol­ecular arrangement of the two mol­ecules in the asymmetric unit. In order to display the extent to which the two mol­ecules in **ZAGZUF** (de Meijere *et al.*, 2014 ▸) agree or disagree in their diastereoisomeric characteristics, Fig. 8 ▸ was drawn using only the command ‘flexibility’ in generating the overlay image.

### Example 4

**ZUDNAP**, space group *P*2_1_2_1_2_1_; [*N*-({2-[(1-benzyl-4-fluoro­prol­yl)amino]­phen­yl}(phen­yl)methyl­ene)alaninato]ni­ckel(II) (Tatum *et al.*, 2015 ▸).

**Chirality data**: Ni1(*S*), C2(*S*), N1(*S*), C4(*S*), and C26(*S*); Ni2(*S*), C30(*S*), N4(*S*), C32(*S*), and C54(*S*).

**Center of mass:**: 0.5000, 0.5000, 0.5000; it is accidentally remarkably close to an inversion center.

**Figure 9:**
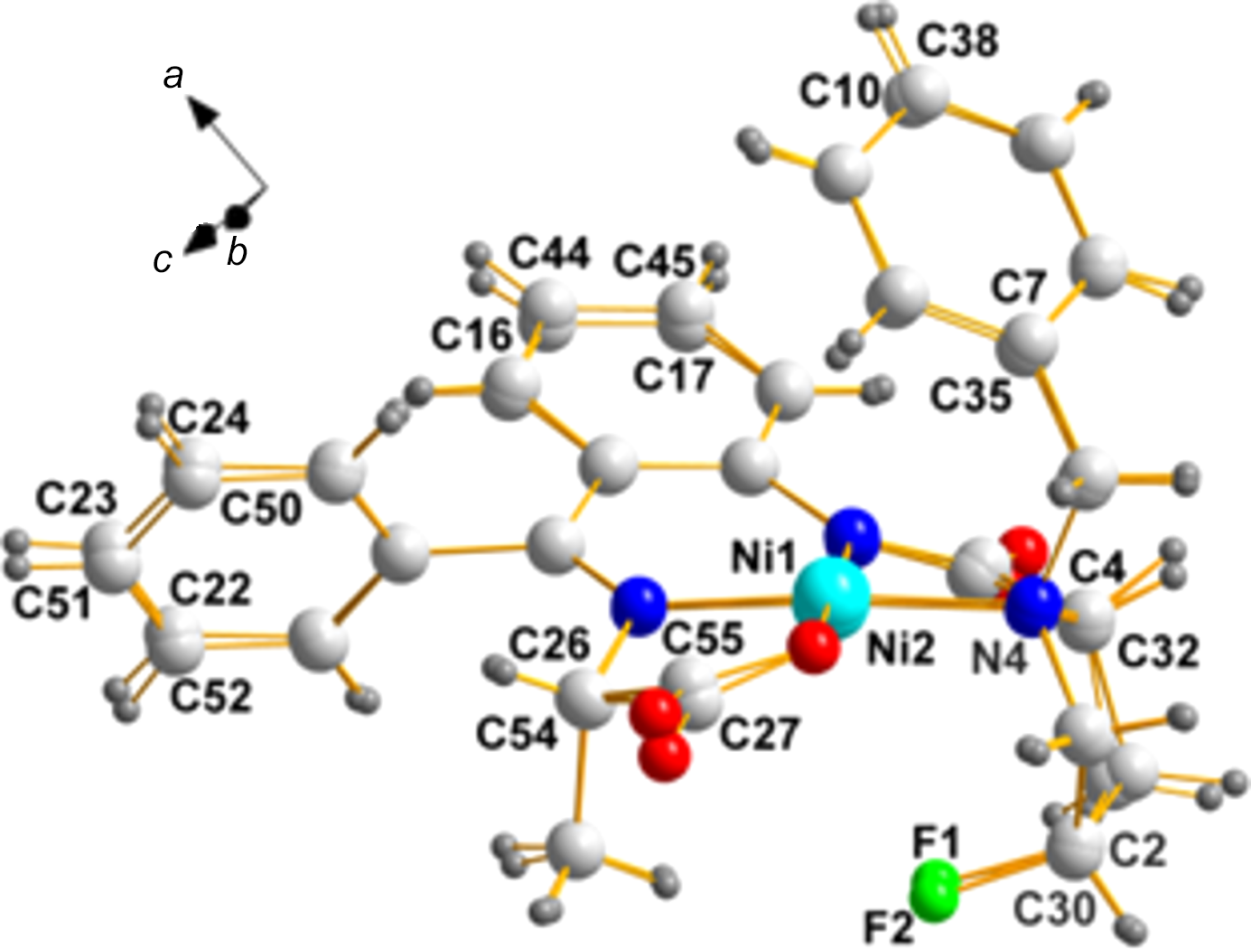
A superposition of the two mol­ecules of the same chirality in **ZUDNAP**. With the exception of N1, the chiral pair sites listed above (C2/C30, C4/C32, and C26/C54) are labeled in this view. Atom N1 is behind N4 and, therefore, its label cannot be displayed.

Fig. 9 ▸ shows the overlay diagram of the two mol­ecules in the asymmetric unit. Another inter­esting species in the same publication (Tatum *et al.*, 2015 ▸) in the space group *P*2_1_2_1_2_1_ is ZUDNET. We leave this exercise to readers who are inter­ested to see how to carry out this process.

Having presented the stereochemical details of our four nickel mol­ecules needed for the discussion below, we now take up an important topic concerning the presence of so many *pseudo-inversion* centers in the packing of these mol­ecules we have labeled as ‘racemic mimics’ – the Flack parameter *x*. Why? Because the crystals **must** belong in the Sohncke class since (*a*) they were prepared with chiral ligands; (*b*) their absolute configuration was retained; (*c*) they are in identical pairs in the lattice; and (*d*) they are located at a variety of pseudo-inversion centers which mimic true ones of proper higher-symmetry centric space groups. It is imperative to determine whether this very valuable criterion is fooled by such circumstances. We retrieved from the CSD (Li *et al.*, 2015 ▸) the appropriate Flack *x* values from each of the four mol­ecules above using the supporting information files from the CIF (see Table 2 ▸).

Given the quality of the refinement in those four examples, it is very satisfactory that the Flack *x* values are those expected for a ‘correct’ space-group assignment, despite the above observations concerning the near centrosymmetric distribution of mass in these diffraction sets. This is fundamental in establishing the validity of our suggestions concerning the characteristics expected from examples of racemic mimic behavior; but, at the same time, we should recall that:

(*a*) in the original recognition of the existence of the phe­nomenon (Furberg & Hassel, 1950 ▸; Schouwstra, 1973*a* ▸, 1973*b* ▸; Mostad *et al.*, 1975 ▸), the quoted authors based most of their observations on the ‘near identity’ of the unit-cell constants of the racemic and enantiopure forms. This is a valuable tool for the searches we suggested above, in which we were able to partially document only once; hopefully, this will be remedied soon with additional examples of a variety of classes of com­pounds for which both members of a pair are structurally known with equally high precision.

(*b*) inter­estingly enough, Furberg & Hassel (1950 ▸) indicated that the pure chiral material seemed to crystallize ‘*as if a twin resembling in its packing that of the true racemate*’. Here is an early recognition that something like the Flack *x* would give validity of the space group assignment.

## Conclusions

Given the values listed above for the four examples under consideration, we conclude that (*a*) yes, the Sohncke space group assignment was correct despite the nearly centrosymmetric distribution of mass in all cases; (*b*) this, also, in spite of the fact that all four cases have an anomalous scatterer (Ni) to ensure that the anomalous contribution is substantial.

So, we suggest that, initially, searches be carried out for cases in which chiral moieties crystallize with *Z*′ = 2.0 and hope that the Notes in the CSD provide examples of the same species crystallizing in a proper supergroup with *Z*′ = 1.0 while having nearly identical unit-cell constants. In such a case, there is a good chance the two crystals are likely to constitute a racemic mimic pair.

## Figures and Tables

**Table 1:** A com­parison of the unit-cell constants of **IHEKIP** and **BOBHIK**

CSD refcode	*a* (Å)	*b* (Å)	*v* (Å)	*V* (Å^3^)
**IHEKIP**	18.511 (5)	18.511 (5)	15.698 (6)	2689.514
**BOBHIK**	18.331 (<1)	18.331 (<1)	15.512 (1)	2606.268

**Table 2:** Flack *x* values for examples 1–4

Example	CSD refcode	Space group	Flack *x*	*R* factor	*T*(K)
1	**NOWPIA**	*P*2_1_	0.033 (7)	4.85	296
2	**WALJOK**	*P*2_1_	0.006 (7)	3.73	296
3	**ZAGZUF**	*P*2_1_2_1_2_1_	−0.032 (14)	2.53	100
4	**ZUDNAP**	*P*2_1_2_1_2_1_	−0.022 (7)	3.64	120

**Table 3:** 

**(*a*) flexibility**	**(*b*) inversion**	**A circle for the image you want**
Leave blank	Leave blank	Use original coordinates in CIF
X	Leave blank	Image generated *only* with flexibility
Leave blank	X	Image generated *only* with inversion
X	X	Image generated with both inversion and flexilibity

## Appendix A A step-by-step description of the process we followed in generating the figures in *Mercury*

<!?tpt=-8.3pt>For those unfamiliar with the use of *Mercury*, we give a brief description of how the figures described in this article were derived.

1. Open *Mercury* by clicking on the appropriate CIF file.

2. Select the option *Mol­ecule Overlay*.

3. On the screen that appears, select *Overlay*.

4. A box containing four different ways of obtaining the result appears ▸:

5. You obtain the desired image by clicking on the relevant dot, on the right. *Mercury* gives a variety of choices for the format, such as .jpg, .tiff, *etc*.

6. (*a*) Using *original coordinates in CIF* means that an image will be created using the unmodified coordinates produced by the structure analysis.

(*b*) Using *inversion* means that the coordinates of one of the two fragments will be inverted prior to generating the overlayed image.

(*c*) Using *flexibility* means that the overlayed image will first be generated and modified as follows: if common fragments such as O—C—O differ in angles or N—C—C—N in ethyl­enedi­amine ligands differ in torsion angles, one of the mol­ecules will be modified in an attempt to generate an overall better fit. For the definition of this, see the *Mercury* manual.

(*d*) Using both *inversion* and *flexibility*. This is obvious from the above.
